# Introducing the CONSORT extension to pilot trials: enhancing the design, conduct and reporting of pilot or feasibility trials

**DOI:** 10.1186/s40409-018-0142-2

**Published:** 2018-02-02

**Authors:** Luciana P. F. Abbade, Joelcio F. Abbade, Lehana Thabane

**Affiliations:** 1 Department of Dermatology and Radiotherapy, Botucatu Medical School, São Paulo State University (UNESP – Univ Estadual Paulista), Av. Prof. Mário Rubens Guimarães Montenegro, s/n, Rubião Júnior, Campus UNESP, CEP 18.618-687, Botucatu, SP Brazil; 2 Department of Obstetric and Gynecology, Botucatu Medical School, São Paulo State University (UNESP – Univ Estadual Paulista), Botucatu, SP Brazil; 3 Biostatistics Unit, Father Sean O’Sullivan Research Centre, St Joseph’s Healthcare, Hamilton, ON Canada; 4 Department of Health Research Methods, Evidence, and Impact, McMaster University, Hamilton, ON Canada

## Abstract

This editorial provides a brief overview of the importance of pilot or feasibility trials or studies, the challenges with current practices in their conduct and reporting, an introduction to the Consolidated Standards of Reporting Trials (CONSORT) extension to pilot trials aimed at improving their reporting, along with some key resources on aspects related to pilot and feasibility studies.

## What are pilot studies and why they are important?

A randomized controlled trial (RCT) is considered the best type of design for studying causal links between interventional exposure and outcomes in all clinical areas including treatment of tropical diseases and procedures related to toxinology. Designing and conducting RCTs is a complex undertaking, and it is frequently time-consuming and expensive. Therefore, conducting a feasibility or pilot study/trial prior to the main RCT is often highly recommended so as to enhance the likelihood of success of the main study, and thereby saving time and costs [1]. Pilot or feasibility study can be defined as a small-scale investigation designed to test the feasibility of methods and procedures for later use in the design of a large scale study [2]. Thus, by definition, the term ‘pilot’ or ‘feasibility’ implies an intention for further definitive work in the future [3].

It is important to emphasize that the primary objectives and outcomes of pilot or feasibility study and the main study are different. The pilot or feasibility trial aims to address questions about prevailing uncertainty regarding the processes or aspects of the main study, such as recruitment, randomization, retention, assessment procedures, validity of tools, new methods and implementation of an intervention. On the other hand, a definitive or main trial has a primary objective of determining the efficacy or effectiveness of a new intervention [4, 5].

Another important distinction between a pilot trial and a main RCT is that in the main study we typically use a statistically significant result based on the minimal clinically important difference (MCID) on the primary outcome of interest as the criterion for declaring success for the trial. In contrast, in a pilot trial, researchers need to establish analogous criteria for determining success of feasibility to guide decisions about moving on to the large-scale trial. Such criteria are typically based on the primary feasibility objectives and outcomes [1].

## What are the current challenges with pilot studies?

Although there is an increasing number of pilot/feasibility studies in the literature, there is substantive evidence that the reporting and conduct is very suboptimal [1, 3, 6, 7]. There is, for example: too much emphasis on hypothesis testing; no clear feasibility objectives/outcomes; inadequate descriptions of analytic plans; and no explicit mention that they were actually done to inform the design of future studies [7, 8]. Some reports of pilot trials show that the study is called “pilot” because of the small sample size, or because it was conducted in single center and without the clear intention of advancing to a main study [1]. Most journals have no editorial policies on pilot studies – making it harder for reviewers and editors to make optimal and consistent editorial decisions on manuscripts reporting such studies [3, 9].

## What is the CONSORT extension to pilot trials?

The CONSORT was first published in 1996 and it was last updated in 2010. It is a guideline designed to improve the transparency and quality of the reporting of RCTs, and it was based on the “standard” two-group parallel design [10]. However, there are several variations to the standard trial methodology, and nowadays there are some CONSORT extensions, for example to cluster trials, non-inferiority and equivalence trials and to pilot and feasibility studies. These extensions of main CONSORT were done to help improve the reporting of these trials.

The CONSORT 2010 statement extension to randomized pilot and feasibility trials that are conducted in advance of a future definitive RCT was published in 2016 [11]. The publication of this extension was motivated by the growing number of studies described as feasibility or pilot and by research that identified deficiencies in their reports and conduct.

As mentioned before, pilot/feasibility trials differ from other forms of RCTs in their objectives – their focus is on assessing feasibility rather than effectiveness or efficacy. Therefore, there are some key differences between the main CONSORT statement [10] and CONSORT statement extension to pilot trials [11]:

- First, an adapted CONSORT extension to abstracts of pilot trials checklist is provided with illustration of how to use the checklist.
- Second, the items of standard CONSORT and those of the CONSORT extension to pilot trials are put side-by-side to make it easier for the researcher to identify items that:
  - ○ remain unchanged (items 4a; 4b; 5; 7b; 8a; 9; 10; 11a; 11b; 13b; 14a; 15; 19; 25);
  - ○ have been modified (items 1a; 1b; 2a; 2b; 3a; 3b; 6a; 6b; 7a; 8b; 12a; 13a; 14b; 16; 17a; 18; 20; 21; 22; 23; 24);
  - ○ were added (items 4c; 6c; 19a; 22a; 26); and
  - ○ were deleted (12b; 17b) – because they were deemed not relevant or applicable for pilot or feasibility trials.
- Third, the guideline also provides some explanation and elaboration for each item – to explain why each included item is important with examples of best practices, and why each excluded item is not relevant to pilot trials. Finally, it recommends an adapted flow diagram of progress of the study with the number of participants that were screened, enrollment, intervention allocation, follow up, and assessment and reporting of each feasibility outcome of the study.

The latest versions on the CONSORT statement of RCT and its extensions are available at www.consort-statement.org and the Equator Network website www.equator-network.org.

## Additional resources for improving the conduct and reporting of pilot trials

Table 1 provides a list of some useful resources that researchers can explore for more information about definitions, challenges with reporting, sample size and reporting pilot studies.

**Table 1:** Some key references about different topics related to pilot or feasibility trials

Main topic	Key references
Definitions	[4, 8, 9]
Challenges with reporting	[1, 3, 6, 7, 12–14]
Sample size estimation	[5, 15]
Reporting	[11, 16, 17]

## Conclusions

Pilot or feasibility studies provide a good opportunity to assess feasibility of large full-scale evaluation studies and they can also enhance the success probability of the main study. For enhancing the transparency and quality of pilot/feasibility trials, they should be scrutinized the same way as the main trials: they should be registered, and they should be reviewed for ethics approval like all research studies. All researchers have the ethical, scientific and economic responsibility and obligation to ensure that their findings about feasibility are widely disseminated in a timely manner. Adoption, adherence and enforcement of the CONSORT extension by journals would certainly make it easier for all stakeholders including authors, journal editors, manuscript reviewers, and researchers to enhance their reporting. Many journals have endorsed the CONSORT statement and its extensions, and recommend adherence to these guidelines in their instructions to authors.

## Abbreviations

MCID: minimal clinically important difference
RCT: randomized controlled trial
